# Strain-specific changes in nucleus accumbens transcriptome and motivation for palatable food reward in mice exposed to maternal separation

**DOI:** 10.3389/fnut.2023.1190392

**Published:** 2023-07-26

**Authors:** Simon Benoit, Mathilde Henry, Sara Fneich, Alexia Mathou, Lin Xia, Aline Foury, Mélanie Jouin, Claudine Junien, Lucile Capuron, Luc Jouneau, Marie-Pierre Moisan, Cyrille Delpierre, Anne Gabory, Muriel Darnaudéry

**Affiliations:** ^1^Univ. Bordeaux, INRAE, Bordeaux INP, NutriNeurO, UMR 1286, Bordeaux, France; ^2^Univ. Paris-Saclay, UVSQ, INRAE, BREED, Jouy-en-Josas, France; ^3^Ecole Nationale Vétérinaire d’Alfort, BREED, Maisons-Alfort, France; ^4^CERPOP, UMR1295, Inserm, Université Toulouse III Paul Sabatier, Toulouse, France

**Keywords:** early life stress, sex effect, operant conditioning, mesolimbic circuit, C3H/HeN mice

## Abstract

**Introduction:**

In humans, adversity in childhood exerts enduring effects on brain and increases the vulnerability to psychiatric diseases. It also leads to a higher risk of eating disorders and obesity. Maternal separation (MS) in mice has been used as a *proxy* of stress during infancy. We hypothesized that MS in mice affects motivation to obtain palatable food in adulthood and changes gene expression in reward system.

**Methods:**

Male and female pups from C57Bl/6J and C3H/HeN mice strains were subjected to a daily MS protocol from postnatal day (PND) 2 to PND14. At adulthood, their motivation for palatable food reward was assessed in operant cages.

**Results:**

Compared to control mice, male and female C3H/HeN mice exposed to MS increased their instrumental response for palatable food, especially when the effort required to obtain the reward was high. Importantly, this effect is shown in animals fed *ad libitum*. Transcriptional analysis revealed 375 genes differentially expressed in the nucleus accumbens of male MS C3H/HeN mice compared to the control group, some of these being associated with the regulation of the reward system (e.g., *Gnas*, *Pnoc*). Interestingly, C57Bl/6J mice exposed to MS did not show alterations in their motivation to obtain a palatable reward, nor significant changes in gene expression in the nucleus accumbens.

**Conclusion:**

MS produces long-lasting changes in motivation for palatable food in C3H/HeN mice, but has no impact in C57Bl/6J mice. These behavioral alterations are accompanied by drastic changes in gene expression in the nucleus accumbens, a key structure in the regulation of motivational processes.

## 1. Introduction

In humans, early-life adversity, such as abuse, trauma or neglect may influence the development of child and exerts long-lasting effects on physiological functions and vulnerability to psychiatric disorders, notably depression, anxiety disorders, and substance abuse (1–3). Early-life adversity produces numerous physiological abnormalities including hypothalamic–pituitary–adrenocortical (HPA) axis hyperactivity, low-grade inflammation, and affects brain areas involved in the regulation of cognitive and emotional processes such as the medial prefrontal cortex (mPFC), amygdala, hippocampus, and ventral striatum (4, 5). Additionally, early-life adversity exacerbates vulnerability to obesity in adult subjects (6), and this effect could be at least partially attributed to poor feeding habits and/or exacerbated motivation for high-calorie foods (7, 8). However, despite a large literature on the impact of early-life adversity on neuropsychiatric vulnerability, its effect on food motivation remains less explored (9).

In rodents, chronic maternal separation (MS) has been used as a *proxy* of early-life adversity. MS effects on emotional behaviors have been extensively documented in rats (10, 11). These effects include cognitive impairments, exacerbated anxiety-and depressive-like behavior as well as anhedonia associated with HPA axis alterations (12–14). Perinatal stress, both during prenatal and postnatal period, is also associated with metabolic disturbances (15–17), and a higher sensitivity to diet-induced obesity (18). Finally, a growing body of evidence suggests that early-life stress impairs reward processes (19). While MS has been associated with reduced sucrose preference, suggestive of an anhedonia phenotype (10, 20), early-life adversity was found to exacerbate addictive drug intake in rodents as well as in humans (21, 22). Strikingly, MS can also lead to lasting disruptions of the reward dopaminergic system (18, 23, 24). Thus, mice exposed to MS combined with unpredictable chronic mild stress or social defeat in adulthood showed transcriptomic changes in the ventral tegmental area and in the nucleus accumbens (NAc) (25, 26). However, the impact of MS on mice motivation for palatable food is still unknown. This is an important issue given that reward circuit alterations have been recurrently reported in the literature in both animal models and humans exposed to early-life adversity (24).

The aim of the present study was then to determine the impact of MS on motivation for palatable food in female and male mice offspring. Since mice are particularly resilient to early-life stress procedures (27, 28), we studied the effects of MS in C57Bl/6J and C3H/HeN mice, two mouse strains used for MS paradigms (25, 29–31). To further characterize the brain changes associated with MS, we also examined gene expression in the mPFC, the NAc, and the hypothalamus, brain areas sensitive to stress and playing a critical role in the regulation of reward processes.

## 2. Materials and methods

### 2.1. Animals

**Figure 1:**
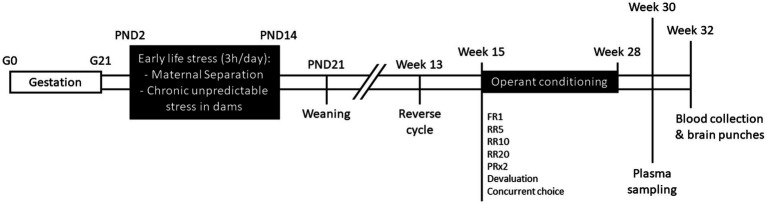
Schematic representation of the experimental timeline. G, gestational day; PND, post-natal day; FR1, fixed-ratio 1; RR5, random-ratio 5; RR10, random-ratio 10; RR20, random-ratio 20; PRx2, progressive-ratio x2.

All experiments were carried out in accordance with French legislation (Directive 87/148, Ministère de l’Agriculture et de la Pêche) and European (Directive 2010/63/EU, 2010 September 22th) and approved by Institutional Regional Committee for animal experimentation (agreement #5012050-A). C57Bl/6J and C3H/HeN mice were obtained from Janvier Labs (Le Genest, Saint-Isle, France) and housed under standard laboratory conditions (23 ± 1°C; 12 h/12 h light/dark cycle; lights on at 7 a.m.; food and water *ad libitum*). After 1 week of habituation, two nulliparous female mice (11 weeks old) were placed with one male from the same strain during 1 week for breeding and then pregnant dams were single-housed in polycarbonate cages (48 × 26 × 21 cm) throughout gestation and lactation. The day of delivery was designated postnatal day 0 (PND0). At PND1, pups from all litters were pulled, sexed, and weighed. Two litters with abnormal number of pups (<3) or sex-ratio (only females) were excluded from the study. Litters were assigned to Maternal Separation (MS, C57Bl/6J, *n* = 7; C3H/HeN, *n* = 7) or control (C57Bl/6J, *n* = 6; C3H/HeN, *n* = 6) groups. The timeline of the experiment was shown in Figure 1.

### 2.2. Maternal separation combined with chronic unpredictable maternal stress

MS was carried out from PND2 to PND14 (180 min daily) (12, 31) and started randomly at 8:30, 9:00, 9:30, 10:15, 10:30, or 11:00 to minimize habituation. During separation sessions, pups were individually separated and kept at 32°C ± 2. During the separation, dams were exposed to a chronic unpredictable stress protocol (PND2: no bedding; PND3: sodden bedding; PND4: tilted cage 45°; PND5: soiled rat bedding; PND6: sodden bedding; PND7: no bedding; PND8: tilted cage 45°; PND9: no bedding; PND10: tilted cage 45°; PND11: sodden bedding; PND12: no bedding; PND13: soiled rat bedding; PND14: forced swim test) [adapted from Franklin et al. (29) and Rincel et al. (31)]. Control pups were left undisturbed with the dams. All pups were weaned on PND21 and grouped in 4–6 mice per cage by same strain, same sex, and same MS condition.

### 2.3. Behavioral assessment in operant chambers

At least 2 weeks before the beginning of the behavioral test, the light/dark cycle was inverted (lights off at 7 a.m.) in order to study mice behavior during the active phase of their cycle. Experiments were conducted during the dark phase between 09:00 and 17:00 h. Male and female offspring’s food motivation for a palatable reward (10% condensed milk in water, 3.25 kcal/g), was assessed at 4–5 months of age in daily 60-min sessions (5 sessions per week) in operant chambers (Imétronic, Pessac, France) equipped with two levers, as previously described (32, 33). For the habituation and the initial training on fixed-ratio 1 (FR-1), mice were food restricted to 85% of their body weight. Then, animals were fed *ad libitum* throughout the experiment except for the concurrent choice test. *Ad libitum* access to food schedule was used in order to examine motivation for palatable food reward independently of the homeostatic state of the animals. Male and female cohorts were tested separately.

#### 2.3.1. Habituation to the apparatus

Mice were placed into the operant chambers without lever for 30 min and milk reward delivered in the drinking cup every 60 s interval. A dose of milk was distributed only when the previous one had been consumed.

#### 2.3.2. Fixed-ratio 1

Mice were initially trained to press one of the two levers (= active lever) on a fixed-ratio 1 (FR-1) schedule. Active lever press resulted in fluid (15 μL of milk solution) delivery associated with a 4 s cue (light above the lever) stimulus presence, in 60-min daily sessions. Responses on the second lever (= inactive lever) were not associated with rewards and were recorded as a measure of non-specific activity. Mice received four FR-1 sessions under mild food deprivation, then mice were tested for an additional FR-1 session in *ad libitum* conditions.

#### 2.3.3. Random-ratio

After FR-1, mice were submitted to random-ratio schedules (RR) in 60-min daily sessions: RR5 (12 sessions), RR10 (3 sessions), and RR20 (12 sessions), with, respectively, a probability of 1/5, 1/10 or 1/20 to be reinforced after one press on the active lever. RR schedules lead to reinforcement following an unpredictable average number of responses per rewards and result in high and consistent response rates that exceed those obtained with FR or interval schedules.

#### 2.3.4. Progressive-ratio

Following training under RR20 schedule, mice were under a progressive ratio (PR) schedule for 2 daily sessions to assess the motivation for the palatable reward. During PR, the number of lever presses required to earn the next reward increased progressively and is multiplied by 2 (2, 4, 8, 16, 32 etc.) For each reinforced response, the animal received sweetened milk (15 μL). The cumulative active lever presses throughout 60 min the session and the total number of active lever presses until a 3 min cut-off without any presses were used as index of motivation. Two animals with very high values (one MS and one Control) were detected as statistical outliers using the Grubb’s test and were removed from analyses.

#### 2.3.5. Devaluation extinction test

Devaluation test allows assessing alteration of the representation of the outcome value. In this test, mice were pre-fed *ad libitum* to either milk or soy-bean oil emulsion Intralipid for 30 min in their home-cage. Immediately after, they were placed into the operant chamber to conduct a lever-press test in a 5-min extinction session. Although both levers were introduced, no reward was distributed. Then, mice received 2 days retraining using a RR20 procedure with free access before the second test. This second session was conducted as previously described, however this time, mice were pre-fed with the alternative outcome. The distribution order of the reward is randomly alternated for each mouse from one session to another.

#### 2.3.6. Concurrent choice

Following one session under RR20 schedule with a food deprivation, a concurrent choice 60-min session test was conducted. In this task, fasted mice could press lever for milk (highly palatable), but their standard lab chow was also available on the floor of chamber opposite to the location of the lever. The total number of lever presses and the amount of lab chow consumed were recorded.

### 2.4. Plasma corticosterone

After the completion of the behavioral assessment, two blood samples were collected, one at the beginning of the active phase and one at the beginning of the inactive phase to assess circadian variation of plasma corticosterone levels. Blood was collected by tail nick using EDTA-coated tubes, were centrifuged (4,000 rpm, 4°C) for 10 min and stored at −20°C until use. Plasma corticosterone levels were determined with an in-house radioimmunoassay using a highly specific antibody as previously described (12). Cross reactivity with related compound such as cortisol was less than 3%. Intra-and inter-assay variations were less than 10% and less than 15%, respectively.

### 2.5. Metabolic hormones assay

Plasma resistin, leptin and insulin levels were measured in 12 h-fasted mice using a Mouse Metabolic Hormone Magnetic Bead-based immunoassay kits (Milliplex MAP Mouse Metabolic Magnetic Bead Panel, cat# MMHMAG-44 K-03, Millipore, Molsheim, FR) according to the manufacturer instructions on samples obtained at the sacrifice. Some samples were not included for technical issues (lack of plasma, out of range values).

### 2.6. Culling and samples collection

Mice were 12 h-food deprived and then were deeply anesthetized with isoflurane and sacrificed by decapitation. Blood was collected for metabolic hormones assessment. Whole brains were collected, medial prefrontal cortex (mPFC) and hypothalamus (HT) were dissected; whereas nucleus accumbens (NAc) was punched on frozen slices and store at −80°C until use.

### 2.7. Microarrays

Total mRNA was extracted from mPFC, NAc, and hypothalamus using a TRIzol extraction kit (Invitrogen) according to the manufacturer’s instructions. RNA concentration, purity and integrity were determined using a ND-1000 spectrophotometer (Nanodrop Technologies, Wilmington, DE, United States) and a bioanalyzer (Agilent, Les Ulis, France) (31). Gene expression profiles were performed at the GeT facility^1^ using Agilent Sureprint G3 Mouse microarrays (8x60K, design 074809) following the manufacturer’s instructions. For each sample, Cyanine-3 (Cy3) labeled cRNA was prepared from 200 ng (mPFC/HT) or 40 ng (NAc) of total RNA using the One-Color Quick Amp Labeling kit (Agilent Technologies, Santa Clara, CA) according to the manufacturer’s instructions, followed by Agencourt RNAClean XP purification (Agencourt Bioscience Corporation, Beverly, Massachusetts). Dye incorporation and cRNA yield were checked using Dropsense™ 96 UV/VIS droplet reader (Trinean, Belgium). Six hundred ng of Cy3-labeled cRNA were hybridized on the Agilent SurePrint G3 Mouse GE microarray slides following the manufacturer’s instructions. Immediately after washing, the slides were scanned on Agilent G2505C Microarray Scanner using Agilent Scan Control A.8.5.1 software and fluorescence signal extracted using Agilent Feature Extraction software v10.10.1.1 with default parameters.

Microarray data were analyzed using R (34) and Bioconductor packages (v 3.0)^2^ (35) as described in GEO accession. Hierarchical clustering was performed using Pearson’s correlation coefficient as distance function and Ward as linkage method. Partial least squares-discriminant analysis (PLS-DA) was performed for each tissue, with group (Ctrl or stress) as a Y response, using *ropls* package (36). Transformed signals were mean-centered and divided by the standard deviation of each variable. A model was fitted using the *limma lmFit* function (37). Pair-wise comparisons of biological conditions were applied using specific contrasts. Probes with Benjamini–Hochberg (BH) false discovery rate (FDR) < 0.05 were considered to be differentially expressed between conditions. Volcano plots were constructed with the *ggplot* function of the R *ggplot2* package. The differentially expressed gene datasets were uploaded into Ingenuity Pathway Analysis software (Qiagen IPA, content version 28,820,210) and a core analysis was performed, with the Agilent SurePrint G3 Mouse GE microarray as background. The canonical pathways with BH *p*s < 0.05 only and the upstream regulators with no flag “bias,” activation z-score < −2 or > 2 and value of p of overlap < 0.05 only were considered. Another analysis was realized using ConsensusPathDB-mouse (38), with the differentially expressed gene list vs. the *mus musculus* database. KEGG pathways with a *q*-value < 0.05 were considered. For TLDA, analysis was performed with the R statistical software (34). For each gene, we compared the expression values within each maternal group in males and females, in C3H/HeN and C57Bl/6J apart. Pair-wise comparisons of maternal groups were conducted using a permutation test, as implemented in the oneway_test function of the *coin* package in R. For each set of tests (i.e., all tested genes for a given pair of maternal groups), *p*-values were BH adjusted for multiple testing. Differences were considered significant when *p*-adj < 0.05. The TLDA data clustering was performed using the ClustVis web tool for visualizing clustering of multivariate data^3^ (39). Microarray data and experimental details are available in NCBI’s Gene Expression Omnibus (40) and are accessible through GEO Series accession number GSE222781.

### 2.8. TaqMan low-density arrays

The top genes identified by IPA were selected for gene expression validation by TaqMan low-density arrays (TLDAs, Applied Biosystems): 44 genes for the *canonical pathway*; 21 genes for the *network (neurological and psychological diseases)*; 9 genes for *nervous system development and behavior*; and 13 genes for *behavior*. All samples were treated with DNase. The experiment was performed on the @BRIDGE platform (INRAE, Jouy-en-Josas, France) according to the manufacturer’s instructions. Four samples were run on each TLDA card in simplicate. Each sample reservoir on the card was loaded with 100 μL of the reaction mix: cDNA template (600 ng) mixed with TaqMan Gene Expression Master Mix (Ap-plied Biosystems). After centrifugation (twice 1 min at 1200 rpm, Heraeus Multifuge 3S Centrifuge), the wells were sealed with a TLDA Sealer (Applied Biosystems). PCR amplification was performed on the 7900HT Real-Time PCR System (Applied Biosystems) using SDS 2.4 software with standard conditions: 2 min 50°C, 10 min 94.5°C, 30 s 97°C (40 cycles), 1 min 59.7°C. Threshold cycle (Ct) values were calculated with the ExpressionSuite v1.0.3 software (Applied Biosystems). The detection threshold was set manually for all genes and was the same for each assay in all tissues. Ct = 39 was used as the cut-off above which, expression level was set to 0. On the TLDA array, 96 genes were studied for each sample: 87 target genes and 9 reference genes (Supplementary Figure S2). Target genes were chosen among the differentially expressed genes found in the microarray experiment in different categories of pathways and biological function. Six on the 9 reference genes were defined as the best reference, using the GeNorm software (41). For each sample, Ct[ref] was the mean of the three Ct values of the reference genes. Then, expression level of target genes was calculated as 2 − (Ct[target gene] − Ct[ref]), as previously described (42).

### 2.9. Statistics

All data are expressed as the means ± SEM (standard error of the mean). Statistical analyses were performed with Statistica 6.0 (StatSoft, Tulsa, OK, United States) and visualized in Prism 9.0 (GraphPad Software, San Diego, CA, United States). Normality and homogeneity of variances were assessed using the Shapiro–Wilk test and Levene test, respectively. Statistical outliers were detected using Grubb’s test. Body weight, hormones, and progressive ratio data were analyzed using a non-parametric Mann–Whitney U test. Behavioral data were analyzed using three-way or four-way ANOVAs with repeated measures with Strain (C57Bl/6J, C3H/HeN) and MS (Control, MS) as between factors and Lever (Active, Inactive), Session (1,2,3,4,5), RR (5,10,20), Devaluation (Non-devaluated, Devaluated) or Concurrent choice (Milk, Milk+Chow) as within factors. When significant interactions were detected, specific comparisons between groups were tested by planned comparisons. “n” corresponds to the number of individuals. *p* < 0.05 was considered statistically significant.

## 3. Results

### 3.1. Chronic maternal separation impairs C3H/HeN mice’s body weight

**Figure 2:**
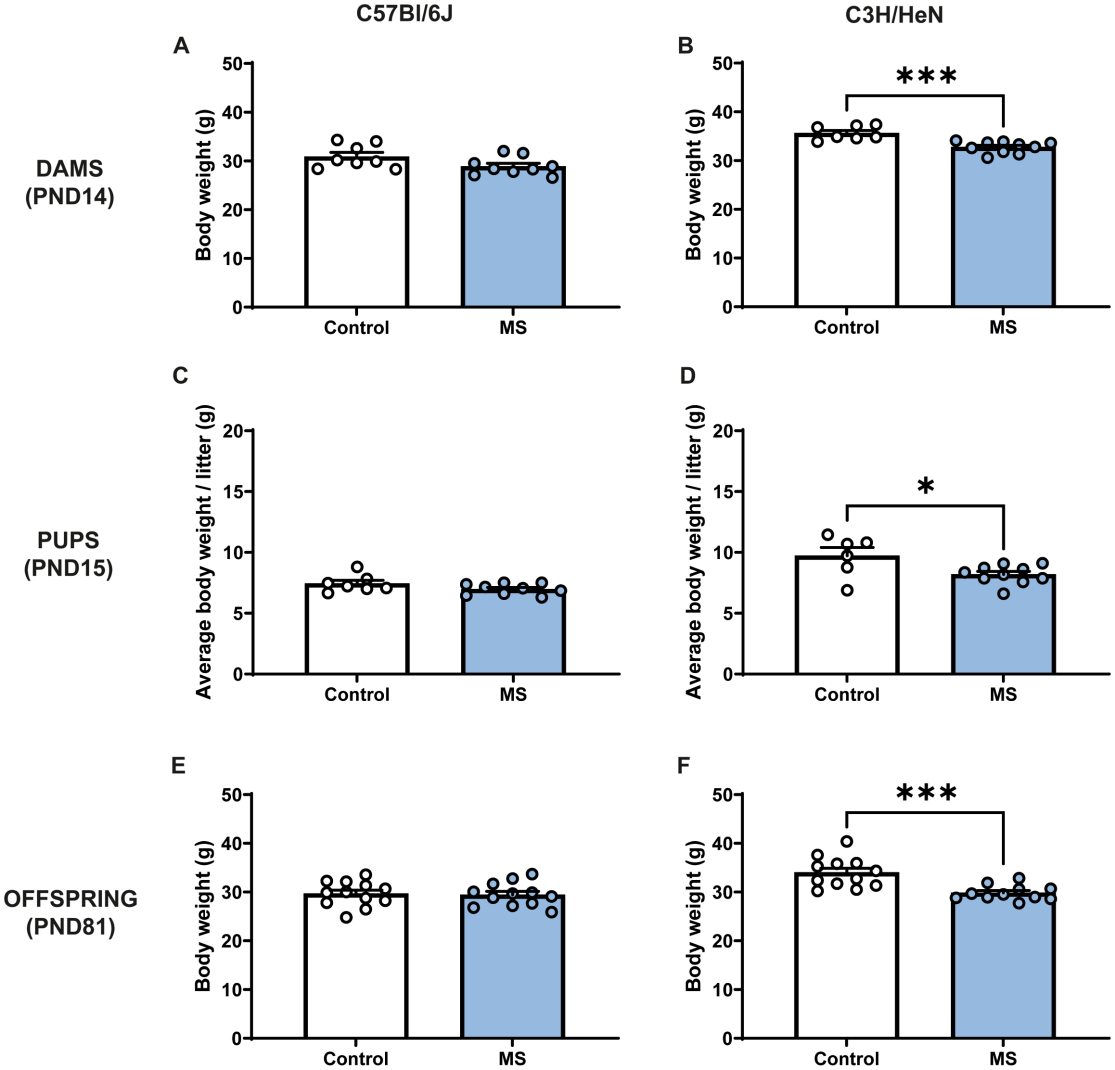
Impact of maternal separation on C57Bl/6J and C3H/HeN dam’s and offspring body weight. Maternal separation decreases body weight of C3H/HeN in both dams and male offspring. Dams’ body weight at PND14: **(A)** C57Bl/6J and **(B)** C3H/HeN. Male pups’ body weight at PND15: **(C)** C57Bl/6J and **(D)** C3H/HeN. Male mice body weight at PND81: **(E)** C57Bl/6J and **(F)** C3H/HeN. *n* = 6–10 per group in dams and in pups; *n* = 12 per group at PND81. Mann–Whitney tests, *p < 0.05, ***p < 0.001.

MS procedure tended to decrease C57Bl/6J dams’ body weight (Mann-Withney test, *U* = 16; *p* = 0.059), whereas stressed C3H/HeN dams displayed ~8% lower body weight compared to the undisturbed C3H/HeN dams (*U* = 1.5, *p* < 0.001) (Figures 2A,B). Similarly, at PND15, MS had no impact on male C57Bl/6J offspring body weight (*U* = 20, n.s., Figure 2C), while MS C3H/HeN male pups exhibited ~16% of decreased in their body weight compared to the control group (*U* = 11, *p* < 0.04, Figure 2D). MS C57BL/6J mice did not differ from controls in adulthood (*U* = 66, n.s., Figure 2E). The effect of MS on C3H/HeN male pups body weight was maintained at adulthood (*U* = 14, *p* < 0.001). MS C3H/HeN mice showed ~12% of decrease in their body weight in comparison to controls. In females, a similar effect of MS was observed in offspring at PND15 (Supplementary Figures S1A,B), but it was not maintained at adulthood (Supplementary Figures S1C,D).

### 3.2. Impact of maternal separation on corticosterone and metabolic hormones plasma levels in C57Bl/6J and C3H/HeN strains

**Figure 3:**
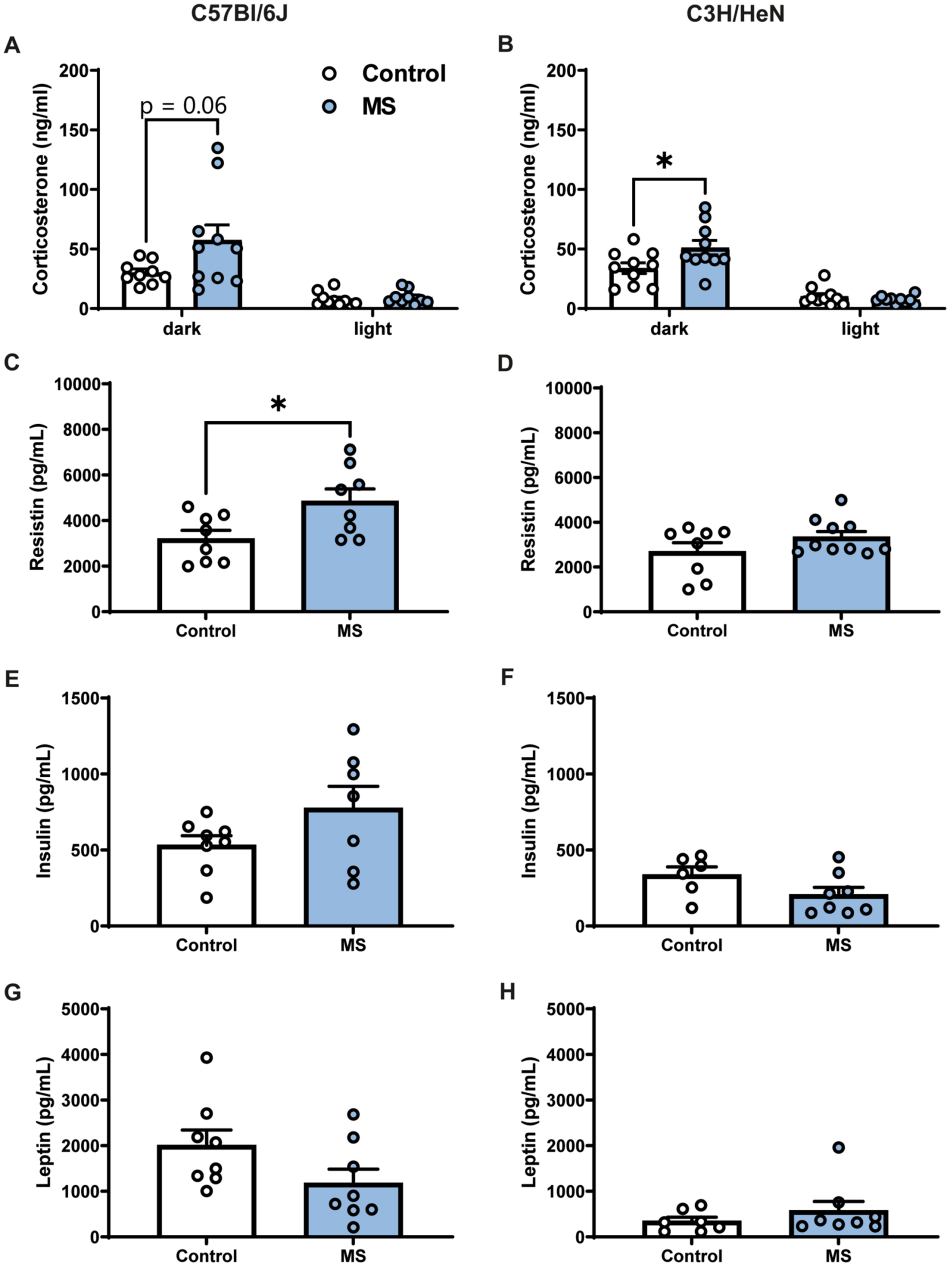
Impact of maternal separation on plasma corticosterone and metabolic hormones levels in C57Bl/6J and C3H/HeN male mice. Plasma corticosterone levels are elevated during the dark phase of the cycle compared to the light period in both **(A)** C57Bl/6J and **(B)** C3H/HeN groups. MS tended to exacerbate the rise of corticosterone during the dark period in C57Bl/6J mice. MS C3H/HeN group had higher plasma corticosterone levels than Control C3H/HeN group during the dark phase. Plasma levels of metabolic hormones in C57BL/6J and C3H/HeN mice: **(C,D)** Resistin levels were significantly increased by MS in C57Bl/6J mice, but not in C3H/HeN mice; **(E,F)** insulin and **(G,H)** leptin levels were not affected by MS. *n* = 6–10 per group. Mann–Whitney tests, *p < 0.05.

The impact of MS on corticosterone and metabolic hormone plasma levels was studied in adult mice (Figure 3). Plasma corticosterone levels were determined at the beginning of the dark and light phases. During the dark period, C57Bl/6J MS male mice tended to have higher plasma corticosterone levels compared to C57Bl/6J controls (*U* = 19, *p* = 0.062, Figure 3A), but the effect of MS was significant in the C3H/HeN strain (*U* = 23, *p* < 0.05, Figure 3B). Indeed, MS C3H/HeN mice exhibited a ~150% increase in corticosterone plasma levels relative to levels in the control group. In contrast, during the light phase, regardless of strain, MS did not affect plasma corticosterone levels (C57Bl/6J, *U* = 27, n.s.; C3H/HeN, *U* = 41, n.s.). A similar profile was reported in females, but the MS effect on corticosterone levels was shown in both strains (Supplementary Figures S1E,F). Regarding metabolic hormone levels, MS significantly increased plasma resistin levels in the male C57Bl/6J strain (*U* = 13, *p* < 0.05, Figure 3C), but no difference was observed in the male C3H/HeN strain (*U* = 31, n.s., Figure 3D). Finally, MS had no effect on plasma insulin and leptin levels in males from all strains (all U > 11, n.s., Figures 3E–H).

### 3.3. Chronic maternal separation exacerbates the motivation for palatable food of C3H/HeN mice fed *ad libitum*

**Figure 4:**
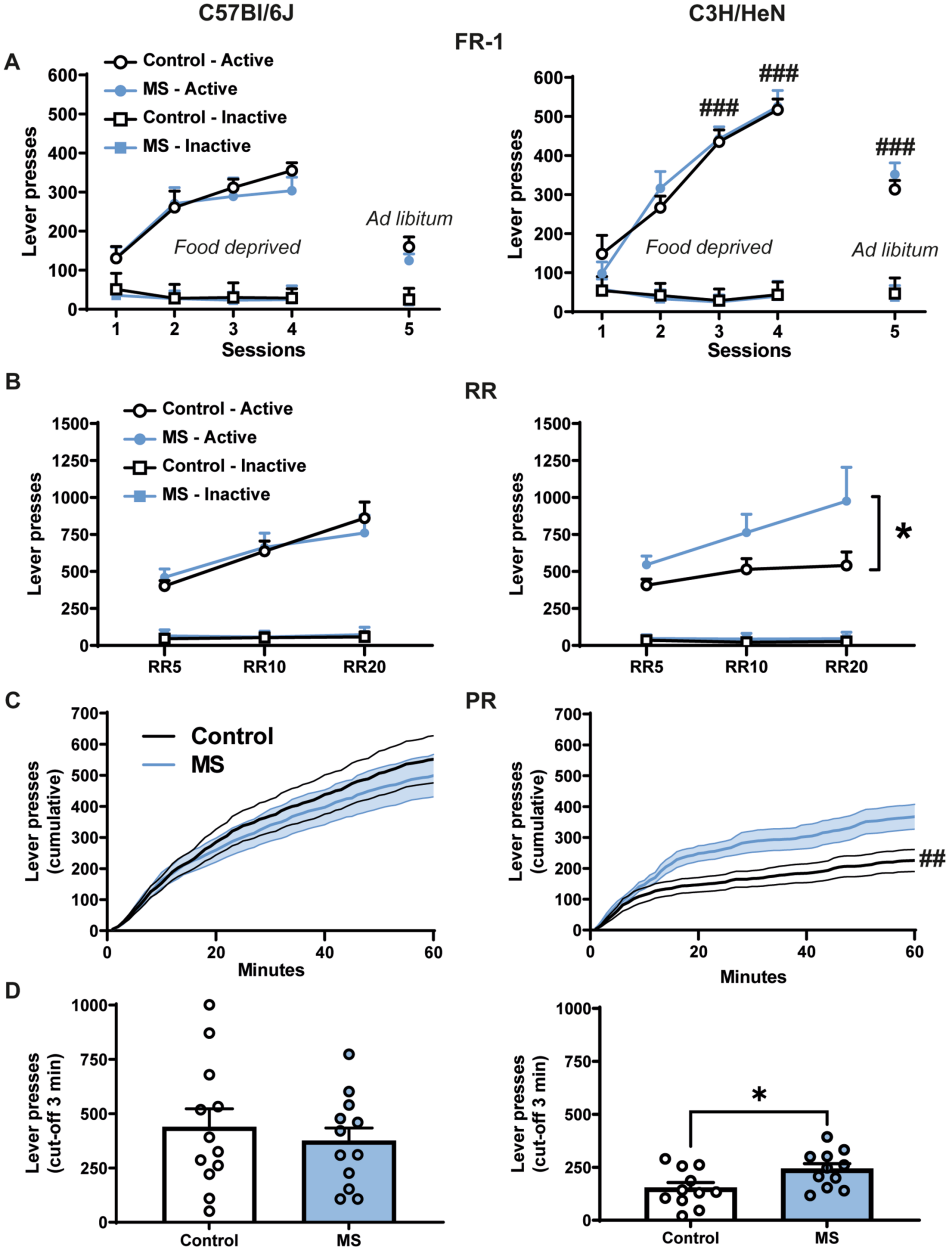
Impact of maternal separation on motivation for palatable food in C57Bl/6J and C3H/HeN male mice. **(A)** There is no impact of MS on mice behavior for the FR-1 schedule. Food deprived C7Bl/6J and C3H/HeN mice progressively increased their number of active lever presses during the 4 sessions in FR-1 schedule. Active lever presses were maintained in *ad libitum* fed animals during the 5th session. Throughout the FR-1 schedule, the number of lever presses on the inactive lever was low. *n* = 12 per group. Four-way ANOVA with repeated measures, followed by planned comparisons, ###*p* < 0.001 C57Bl/6J vs. C3H/HeN. **(B)** During the random ratio schedules (RR5, RR10, RR20 *ad libitum* condition), MS increased the number of active lever presses in C3H/HeN mice but had no effect in C57Bl/6J mice. Motivation for palatable food was exacerbated in male C3H/HeN mice submitted to MS. *n* = 12 per group. Four-way ANOVA with repeated measures, followed by planned comparisons, *p < 0.05 Control vs. MS. **(C)** During the progressive-ratio schedule (PR), the increase of the cumulated number of lever presses over a 60-min session differed according the strain. *n* = 11 per group. ANOVA followed by planned comparisons, ##*p* < 0.01 C57Bl/6J vs. C3H/HeN. **(D)** MS C3H/HeN mice displayed higher total active lever presses than control C3H/HeN during the PR (cut-off 3 min); control C57Bl/6J group was similar to the MS C57Bl/6J group. *n* = 11 per group. Mann Whitney test, *p < 0.05 Control vs. MS.

We used operant conditioning paradigm to examine the effect of MS on the motivation for palatable food (Figure 4). On the fixed-ratio 1 (FR-1) schedule, four-way ANOVA with repeated measures reveals significant strain effect [*F*_(1,44)_ = 21.06, *p* < 0.001], lever effect [*F*_(1,44)_ = 545.96, *p* < 0.001] and session effect [*F*_(4,176)_ = 64.142, *p* < 0.001] (Figure 4A and Supplementary statistics). Furthermore, the ANOVA indicates that the number of active lever presses differs across sessions between strains [Lever × Session × Strain effect, *F*_(4,176)_ = 11.09, *p* < 0.001]. In contrast, MS had no significant effect on lever presses in FR-1 whatever the strain considered [MS effect, *F*_(1,44)_ = 0.14, n.s.; Lever × Session × Strain × MS effect, *F*_(4,176)_ = 1.11, n.s.]. In both C57Bl/6J and C3H/HeN strains, the number of active lever presses was higher than the number of inactive lever presses (*p* < 0.001 for each session) and it significantly increased across the session 1 to 4 when animals were tested under fasted condition (*p* < 0.001, S1 vs. S2, S3, S4). C3H/HeN mice displayed a ~150% increase of their active lever presses compared to C57Bl/6J mice during the S3, S4, and S5 sessions (*p* < 0.001). When mice were fed *ad libitum* (5th session under FR1 schedule), the number of presses on the active lever decreased in both strains (*p* < 0.001, S4 vs. S5), but it remained higher than inactive lever (*p* < 0.001). Overall these results indicate that both strains progressively increased their number of active lever presses during the four sessions of FR-1 schedule conducted in fasted animals, this effect was exacerbated in C3H/HeN strain. Active lever presses were maintained in *ad libitum*-fed animals. Throughout the FR-1 schedule, the number of lever presses on the inactive lever was low. Finally, whatever the session, MS did not alter the performances neither in C57Bl/6J, nor in C3H/HeN strain.

A random ratio (RR) schedule with a 1/5, 1/10, and 1/20 probability (RR5, RR10, and RR20, respectively) to obtain one palatable food reward was next performed in *ad libitum* fed animals (Figure 4B and Supplementary statistics). Four-way ANOVA with repeated measures showed significant lever effect [*F*_(2,44)_ = 160.04, *p* < 0.0001] and RR effect [*F*_(2,88)_ = 28.88, *p* < 0.0001], but no main effect of strain [*F*_(2,44)_ = 0.094, n.s.] or MS [*F*_(2,44)_ = 2.6207, n.s]. Importantly, MS differentially affected the number of active lever presses across the RR sessions according to the strain [Strain × RR × Lever × MS, *F*_(2,88)_ = 3.46, *p* < 0.05]. Controls and MS C57Bl/6J mice increased their number of active lever presses according to the probability to obtain the reward (RR5 vs. RR10 or RR20, *p* < 0.0001). In contrast, whereas control C3H/HeN mice were stable across RR session (RR5 vs. RR10 or RR20, n.s.), MS C3H/HeN mice exhibited a significant escalation of their active lever presses (RR5 vs. RR10 or RR20, *p* < 0.0003). MS C3H/HeN mice showed an overall ~160% increment of their active lever presses in comparison to control C3H/HeN mice (*p* < 0.05). A similar profile was reported in females with no impact of MS in C57Bl/6J and a significant increase of lever presses in C3H/HeN mice exposed to MS (Supplementary Figures S1G,H).

To further study motivation, mice were then submitted mice to a progressive-ratio procedure during which the number of lever presses to obtain a single food reward progressively increased. Animals were tested in sated condition. Under a progressive-ratio schedule (PRx2), the cumulative number of lever presses were differentially affected by MS according to the strain across time [Figure 4C, three-way ANOVA, Strain effect, *F*_(1,42)_ = 9.769, *p* < 0.01; MS effect, *F*_(1,42)_ = 0.816, n.s; Time effect, *F*_(59,2478)_ = 141.082, *p* < 0.001; Strain × MS × Time effect, *F*_(59,2478)_ = 2.646, *p* < 0.001]. Control C57Bl/6J mice exhibited higher lever presses than Control C3H/HeN mice (*p* < 0.01), but MS exposure in C3H/HeN mice suppressed this difference. Additionally, while MS had no impact on the number of active lever presses before the cut-off (3 min without any lever presses) in C57Bl/6J mice (*U* = 66; n.s.), MS significantly increased the active lever presses in C3H/HeN mice (*U* = 29; *p* < 0.05) (Figure 4D). Overall, our results indicate that C57Bl/6J mice exhibit a high motivation for palatable food when fed *ad libitum*, and that MS leads to an exacerbation of the motivation for palatable food restricted to the C3H/HeN strain. In females, whatever the strain, MS had no significant impact on progressive ratio results (data not shown).

**Figure 5:**
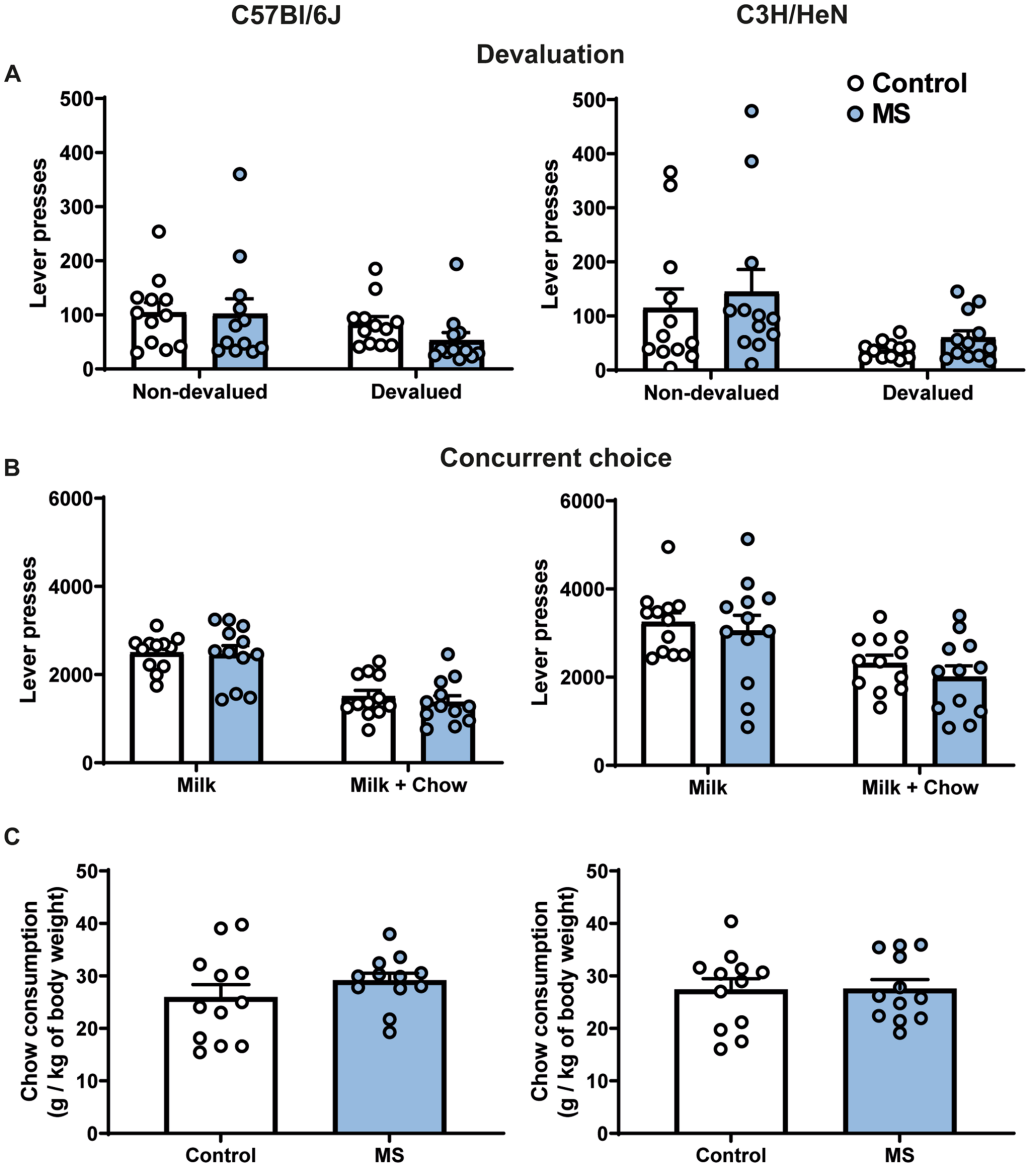
Impact of maternal separation on outcome devaluation and concurrent choice procedures in C57Bl/6J and C3H/HeN male mice. Maternal separation does not affect representation of outcome value and rate of lever presses in the concurrent choice procedure in C57Bl/6J and C3H/HeN mice. **(A)** Lever presses during a 5-min extinction test in a non-devaluated condition (Intralipid consumption before test) and in a devaluated condition (milk consumption before test). **(B)** Lever presses during a concurrent choice procedure: milk was provided as sole reward after instrumental response or standard chow was provided as free alternative to milk reward. Three-way ANOVA. **(C)** Amount of chow consumed during the concurrent choice test. *n* = 12 mice per group. Two-way ANOVA.

In order to determine whether the exacerbated motivation in MS male C3H/HeN was due to an alteration of the representation of outcome value, we studied their performances in a devaluation procedure. In the sensory-specific satiety test, a significant effect of pre-feeding was observed for the number of lever presses, this effect was not affected by MS and was similar between strains [three-way ANOVA, Devaluation effect, *F*_(1,44)_ = 15.138, *p* < 0.001; Strain effect, *F*_(1,44)_ = 0.030, n.s.; MS effect, *F*_(1,44)_ = 0.058, n.s; Devaluation × Strain × MS effect, *F*_(1,44)_ = 0.1271, n.s., Figure 5A]. Finally, in the concurrent choice test (conducted in fasted animals), the presence of an alternative reward less palatable (chow), but freely available in the operant chamber, reduced the number of lever presses for sweetened milk similarly between control and MS groups of both strain [Concurrent choice effect, *F*_(1,44)_ = 10.787, *p* < 0.001; Strain effect, *F*_(1,44)_ = 3.25, n.s., MS effect, *F*_(1,44)_ = 2.46, n.s.; Concurrent choice × Strain × MS effect, *F*_(1,44)_ = 0.316, n.s., Figure 5B]. The total amount of chow consumed during the test was similar between groups in both strains [Two-way ANOVA, Strain effect, *F*_(1,44)_ = 0.0004, n.s.; MS effect, *F*_(1,44)_ = 0.72, n.s., Strain × MS effect, *F*_(1,44)_ = 0.59, n.s., Figure 5C].

### 3.4. Maternal separation modifies the transcriptional profile in the nucleus accumbens of C3H/HeN

**Figure 6:**
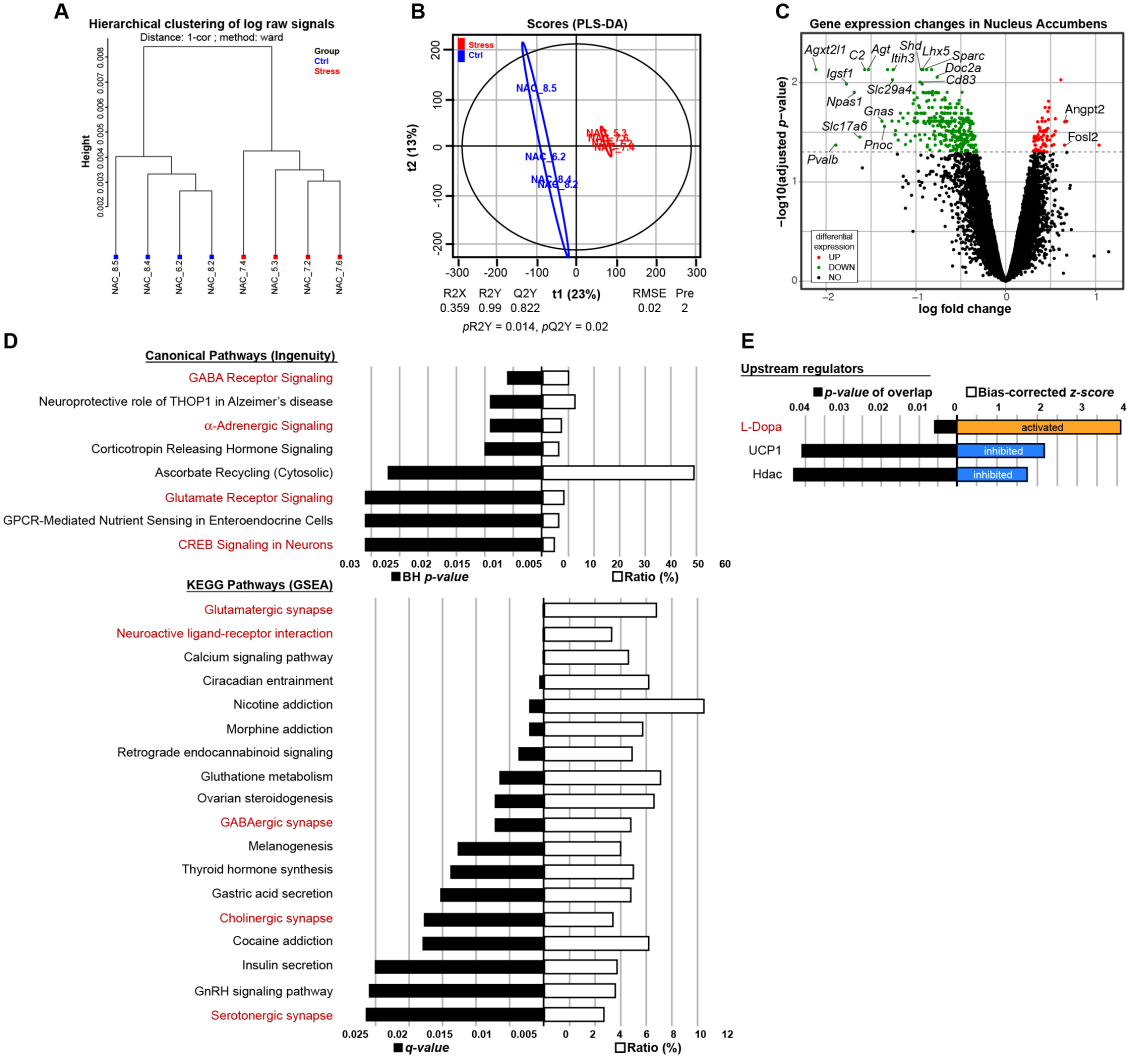
Impact of maternal separation on gene expression in the nucleus accumbens (NAc) of male C3H/HeN mice. Maternal separation drastically modifies the transcriptional profile in the NAc of male C3H/HeN mice. Clustering by Euclidean distance of the raw transcriptomic datasets of NAc from Control (blue) or MS (red) male adult offspring **(A)**. Score plots from the PLS-DA classification into Control (blue) and MS (red) groups. A model is considered robust when the response variance explained (R2Y = 0.99) is higher than the predictive performance of the model (Q2Y = 0.822). A model with a Q2Y > 0.5 is considered to have a good predictive performance **(B)**. Volcano plot depicting significantly differentially expressed genes in the NAc of C3H/HeN mice between the MS and Control conditions **(C)**. Top Canonical Pathways and KEGG pathways **(D)** and top upstream regulators **(E)** involved. In red are depicted the pathways or regulators associated with NAc neuronal function. *n* = 4 mice per group.

To investigate the molecular brain signature associated with exacerbated motivation in MS animals, after the operant task, we performed transcriptomic analysis in male C3H/HeN offspring. We hybridized RNA from whole-tissue micropunches of NAc, mPFC, and hypothalamus on Agilent microarrays. Descriptive analysis via hierarchical clustering showed a clear separation for NAc only (Figure 6A and Supplementary Figures S2A,D). PLS-DA analysis was able to build a model in each tissue but only the model in the NAc was validated (Figure 6B, *p*R2Y = 0.014, *p*Q2 = 0.02 for NAc, Supplementary Figure S2B, *p*R2Y = 1, *p*Q2 = 0.84 for mPCF, and Supplementary Figure S2E, *p*R2Y = 0.78, *p*Q2 = 0.94 for hypothalamus). No differentially expressed genes (DEGs) were found in the mPFC and hypothalamus (Supplementary Figures S2C,F), while 375 DEGs were found in NAc: 306 downregulated and 69 upregulated (Figure 6C and Supplementary Table S1). Therefore, the transcriptional effect is restricted to the NAc tissue. A bioinformatic analysis of functions and networks was carried on the 375 DEGs using the IPA software and the ConsensusPathDB-mouse (CPDB) website. This detected an overrepresentation of 8 canonical pathways with BH *p*s < 0.05 and 18 KEGG pathways with *q* < 0.05, respectively (Figure 6D). Among these pathways, 2 are common to the 2 analyses: GABA receptor signaling/GABAergic synapse and Glutamate receptor signaling/Glutamatergic/synapse. Pathways analysis also pointed out stress related pathways (α-adrenergic signaling and CRH signaling) and addiction pathways (nicotine, morphine cocaine). The IPA analysis also predicted upstream regulators, which may be causing the observed gene expression changes. Interestingly, among the 3 predicted upstream regulators, the L-Dopa had the lowest *p-*value and the highest *z*-score, with a predicted activation whereas the uncoupling protein 1 *Ucp1* and the histone deacetylases *Hdac* were predicted as inhibited (Figure 6E). Finally, using TLDA assays, we performed a RT-qPCR analysis on 84 genes of NAc samples from males and females of both strains. Microarray results obtained in C3H/HeN male mice were validated by TLDA (Supplementary Figure S2G, Spearman correlation *rho* = 0.754; *p* < 2.2e^−16^). A clustering analysis of these TLDA results showed clearly the differential expression of the 84 genes between control and MS C3H/HeN males. In contrast, we did not observe any differential expression for the 84 genes identified in C3H/HeN mice when we compared control and MS C3H/HeN females or control and MS C57Bl/6J regardless of the sex (Supplementary Figure S2H).

## 4. Discussion

MS models in mice have been widely used to examine the long-lasting effects of early-life stress on emotional function. Herein, we report evidence for a strain-dependent effect of MS on the motivation for a palatable nutritional reward in an operant conditioning paradigm. Our data show that MS combined to unpredictable chronic mild stress in lactating dams exacerbated motivation for a highly palatable food reward (sweetened milk) in both male and female C3H/HeN mice, but had no effect in C57Bl/6J strain. The transcriptomic analysis revealed that exacerbated motivation in MS C3H/HeN male mice was associated with marked changes in gene expression in the NAc, whereas no significant changes were reported in the PFC or hypothalamus.

A primary and key result of our study is the overall difference in behavioral and brain gene expression outcomes observed between C3H/HeN and C57Bl/6J strains in response to early life stress. Body weight and food motivation were specifically altered in C3H/HeN but not in C57Bl/6J suggesting that C3H/HeN strain is more susceptible to MS for these studied parameters. This comforts previous findings showing a resilience of the C57Bl/6J strain to the MS procedure effects (27, 43). Indeed, despite the fact that MS procedure has been fairly well-established in rats (10, 44), results in mice especially in the C57Bl/6J strain are very heterogenous. Previous work using an early-life stress paradigm close to our study (3 h MS from PND1 to PND14, coupled to an unpredictable restraint stress or forced swim stress in dams during separation) showed increased depressive-like behaviors, reduced anxiety, but had no impact on serum insulin levels in C57Bl/6J offspring exposed to early stress (29, 45, 46). Regarding the HPA axis function, we demonstrated that MS increases plasma corticosterone at the beginning of the dark phase in both strains, consistent with previous results in the literature (27, 47). This finding is important since it demonstrates that despite differential strains’ sensitivity to MS in food motivation, both strains are affected by MS. Most of previous works conducted in C57Bl/6J exposed to MS report long-term effects on behavior, only when animals are re-exposed to an additional stressor such as early weaning at PND17 (48), chronic social defeat or unpredictable chronic mild stress (UCMS) at adulthood (25, 26). Without additional stress, emotional behaviors in MS C57Bl/6J mice generally do not significantly differ from controls (25–27, 43, 49, 50). On the other hand, C3H/HeN strain displays gut dysfunction after MS (17, 30, 31) and emotional impairments in a multi-hit model combining MS, maternal UCMS and prenatal infection (31). The different susceptibility to MS procedure between the C57Bl/6J and C3H/HeN strains could be due to maternal care differences. Accordingly, C3H/HeN dams display more pups licking and nursing than other strains (51). This robust maternal behavior may be more affected by the disruption of the nest and pups’ separation associated to MS procedure. Additionally, C3H/HeN strain are more anxious and show a higher sensitivity to stress compared to other mice strains; whereas C57Bl/6J strain has been recurrently described in the literature as a strain particularly resilient to stress (52). Taken together, this suggests that MS effects may vary depending of strains and behavioral dimensions.

Clinical literature suggests that early-life adversity is associated with higher risk to develop food addiction behaviors and obesity in adulthood (6, 7, 53). Although emotional behaviors have been extensively studied in MS literature, motivation for food reward has been less explored. Previous works in rodents exposed to early-life stress, demonstrate that MS exacerbates drugs of abuse motivation and ethanol intake (21, 54). Since addictive drugs and palatable food partially share common neurobiological substrates, we hypothesized that MS will affect food motivation and impact the mesolimbic circuit. Previous findings in rats showed that early-life stress exacerbates motivation for palatable food as indicated by their higher breakpoint in operant task and their lower latency to reach chocolate pellets in a runway task (18, 55). Here, we present data supporting an impact of early-life stress on palatable food motivation in operant conditioning test in C3H/HeN mice. This effect is strain-dependent, but it is observed in both male and female MS C3H/HeN mice. Interestingly, differences were reported in RR and PR schedules when the effort to obtain the palatable food reward is high. In contrast, FR-1 schedule performances, which reflect more the ability to learn the task, were similar between control and MS groups. Noteworthy, exacerbated motivation for palatable food in MS animals was found in mice non-submitted to food restriction during the instrumental task. These results suggest that regardless of their nutritional status, stressed animals may be more prone to seek and consume calorie-dense food. Accordingly, MS rats ate more palatable food when they have a free access in their home cage (18, 55, 56). Sweetened milk is highly palatable for mice and sugar has been shown to share numerous behavioral and neurophysiological processes with drug of abuse, suggesting the possibility of sugar addiction (57). Our results are in accordance and extend recent finding showing that a single long lasting (23 h) separation at PND3 in mice produces enhanced binge-eating behavior after repetitive cycles of re-exposure to a high-fat diet in adulthood (49) and clinical literature showing that early-life adversity is associated with a higher risk of developing food addiction behaviors and obesity in adulthood (6, 7, 53). Finally, in the present work, we also showed differences in operant response between C57Bl/6J and C3H/HeN strains. While C3H/HeN strains made higher active lever presses under fasting conditions, they were less motivated than C57Bl/6J strains when fed *ad libitum* in RR and PR patterns. We cannot exclude that these strain differences contribute to the variation in MS susceptibility between strains.

An important finding in the present work is that MS C3H/HeN males exhibiting exacerbated motivation for palatable food had marked changes of brain gene expression (adj *p*-values: 375 genes differentially expressed) specifically in the NAc, a key region for the regulation of motivation and reward processing. Notably, we validated a large amount of gene differentially expressed in MS C3H/HeN males using TLDA. Interestingly none of the genes significantly affected by early-life stress in C3H/HeN mice were changed in C57Bl/6J MS mice. However, given that C57Bl/6J results were not obtained using microarray, we cannot exclude that changes affecting other genes also occur in this group. Previous transcriptomic studies demonstrated a significant impact (with non-adjusted *p*-values) of early-life stress using RNAseq analysis in C57Bl/6 strain (25, 26). Using adjusted *p*-values, we did not detect a significant effect of MS on gene expression in the PFC and hypothalamus, indicating that NAc is a brain area particularly affected by MS in C3H/HeN. The lack of transcriptional change in the hypothalamus is quite surprising given the importance of this brain area in the effects of stress and in the control of food intake. Again, it is important to note that we used here the Benjamini-Hochberg adjusted *p*-values method which is highly conservative and may lead to under detection of change in gene expression between groups. Further studies should be conducted to examine the impact of MS on specific nuclei of the hypothalamus such as the lateral hypothalamus.

In the NAc, we identified several genes such as *Agt, Igsf1, Gnas, Pnoc, Npas1, Pvalb*, or *Fosl2* affected by early-life stress in C3H/HeN male mice which have been previously reported to be modified in the NAc or in the VTA after chronic stress procedures (25, 26). Interestingly, *Gnas* (Guanine Nucleotide-Binding Protein G Subunit Alpha) and *Pnoc* (prepronociceptin) have previously been linked to motivation and reward regulation (58–61). *Angpt2* (angiopoietin-2) plays a role in angiogenesis and its expression is induced by inflammatory markers (62). *Angpt2* has not been linked with motivational changes, but recent data suggest that up-regulation of immune factors within the NAc may correlate with addictive phenotype in rats (63) and compulsive sucrose seeking in mice fed with high-fat diet (64). The mechanisms underlying this marked impact of early-life stress on NAc transcriptome in C3H/HeN mice need to be explored. However, pathways analysis (using IPA and CPDB) on NAc genes revealed that the excitatory (Glutamate receptor signaling) and inhibitory (GABA_A_ receptor signaling) pathways are both affected by early-life stress. GABAergic and glutamatergic systems play a major role in neurodevelopment and disruption of these systems have been involved in numerous neurodevelopmental disorders including autism, schizophrenia or ADHD (65). Furthermore, a large body of evidence has linked various perinatal stress paradigms with altered excitatory/inhibitory balance (66–69). Importantly, major upstream regulators identified by IPA are *L-Dopa*, *Ucp1* and *Hdac* suggesting that these factors may contribute to the effects of early stress. UCP1 (uncoupling protein 1, a proton carrier protein generating heat via non-shivering thermogenesis) has been recently identified in the brain, though its role still remains unclear (70). HDAC is an important epigenetic regulator and its inhibition in the NAc promotes drug self-administration (71). As epigenetic regulator, HDAC may be involved in numerous changes in the NAc transcriptome. Among the upstream regulators, the dopamine precursor L-Dopa may have an important function in the observed effects on motivation for a highly palatable reward. L-Dopa therapy in Parkinson disease has been shown to increase the risk of addictive behaviors, including compulsive eating (72). NAc dopamine is involved in palatable food-seeking behavior during instrumental task for schedules that have high-work requirements such as RR or PR (73). A limitation of the present study is that we cannot conclude a direct effect of MS on the NAc transcriptome that could lead to altered food motivation in C3H/HeN mice. Fasting prior to brain sampling can differentially modify gene expression in the NAc in MS CH3/Hen mice. Furthermore, a recent work demonstrates that operant training for highly palatable food in mice changes translating mRNA in dopaminoceptive neurons of the NAc (74). It is then possible that altered gene expression in the NAc results in part from a different rate of exposure to palatable food in C3H/HeN mice exposed to MS.

In conclusion, our work reveals that early-life stress increases motivation for palatable food in *ad libitum* fed adult C3H/HeN mice. This effect is associated with marked changes in gene expression within the NAc. Importantly, exacerbated palatable food motivation after MS is found in both male and female C3H/HeN mice, but this effect is strain dependent suggesting a relative resilience of C57Bl/6J. Overall, our study confirms that early-life adversity has enduring effects on reward circuits and highlights the importance to further explore the impact of early-life stress on motivational processes, in the context of food overconsumption and obesity.

## Data availability statement

The datasets presented in this study can be found in online repositories. The names of the repository/repositories and accession number(s) can be found at: https://www.ncbi.nlm.nih.gov/, GSE222781.

## Ethics statement

The animal study was reviewed and approved by Institutional Regional Committee for animal experimentation (agreement #5012050-A).

## Author contributions

MD designed and supervised the study. SF, AF, MJ, M-PM, LJ, and AG performed and analyzed the transcriptomics. AM performed the behavioral tests. LX performed the metabolic measures. MH wrote the first draft of the manuscript. SB and MD wrote the manuscript. All authors discussed and commented on the manuscript.

## Funding

This work was supported by the Agence Nationale de la Recherche (ANR-12-DSSA-0004 Incorporation Biologique et Inégalités Sociales de Santé), Fondation Recherche Médicale (ENV-Environnement—Santé; ENV202003011543), Université de Bordeaux and INRAE.

## Conflict of interest

The authors declare that the research was conducted in the absence of any commercial or financial relationships that could be construed as a potential conflict of interest.

## Publisher’s note

All claims expressed in this article are solely those of the authors and do not necessarily represent those of their affiliated organizations, or those of the publisher, the editors and the reviewers. Any product that may be evaluated in this article, or claim that may be made by its manufacturer, is not guaranteed or endorsed by the publisher.

## Footnotes

^1^https://www.genotoul.fr/

^2^www.bioconductor.org

^3^https://biit.cs.ut.ee/clustvis/
