# Supplementary material for: Strain-specific changes in nucleus accumbens transcriptome and motivation for palatable food reward in mice exposed to maternal separation
Source: Front Nutr. 2023 Jul 26;10:1190392. doi: 10.3389/fnut.2023.1190392 (PMC10411197; doi:10.3389/fnut.2023.1190392)
Supplement: Supplementary file 1 [file Data_Sheet_1.zip › Supplementary Figures.docx]

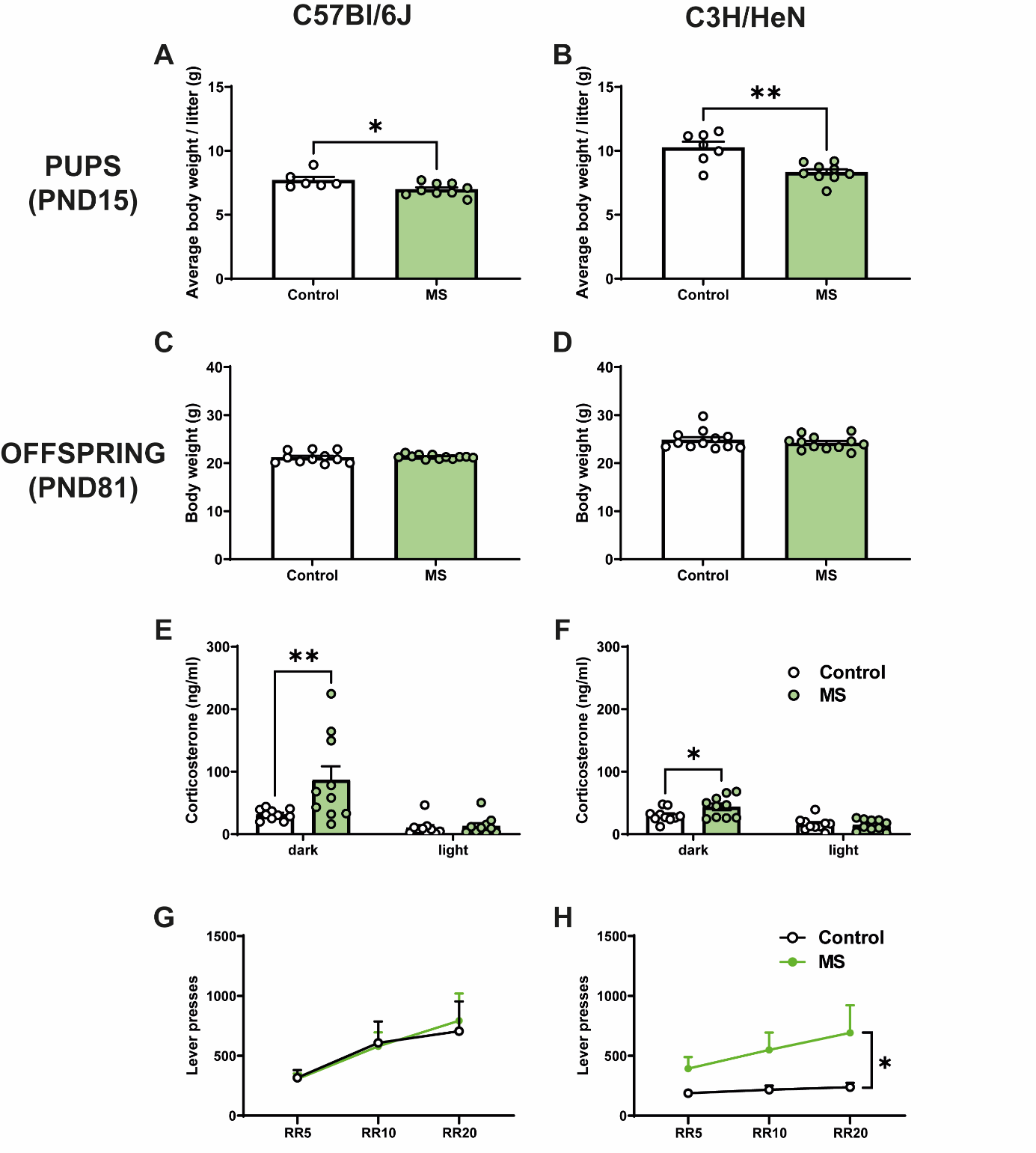


Figure S1. Impact of maternal separation on pup’s body weight, corticosterone level and motivation for palatable food in C57Bl/6J and C3H/HeN female mice. Maternal separation impaired female pups’ body weight at PND15: (A) C57Bl/6J and (B) C3H/HeN. n = 6-9 *per* group, Student t test (C57Bl/6J: t_(13)_ = 2.500, p = 0.0266; C3H/HeN: t_(14)_ = 3.986, p < 0.01). Maternal separation had no effect on females’ body weight at PND81: (C) C57Bl/6J and (D) C3H/HeN. Maternal separation increased plasma corticosterone levels during the dark phase in female mice: (E) C57Bl/6J and (F) C3H/HeN. n = 10 *per* group, Two-way ANOVA followed by Sidak *post-hoc,* C57BL/6J, Period x MS effect, F_(1,13)_ = 5.794, p < 0.05; C3H/Hen, Period x MS effect, F_(1,13)_ = 4.78, p = 0.05, p < 0.05. (G) Control C57Bl/6J and MS C57Bl/6J female mice showed similar lever presses during RR procedure; MS increased lever presses in C3H/Hen female mice compared with controls. n = 8 *per* group, Two-way ANOVA, C3H/Hen, MS effect, F_(1,13)_ = 5.818, p < 0.05. ** p < 0.01, * p<0.05

**
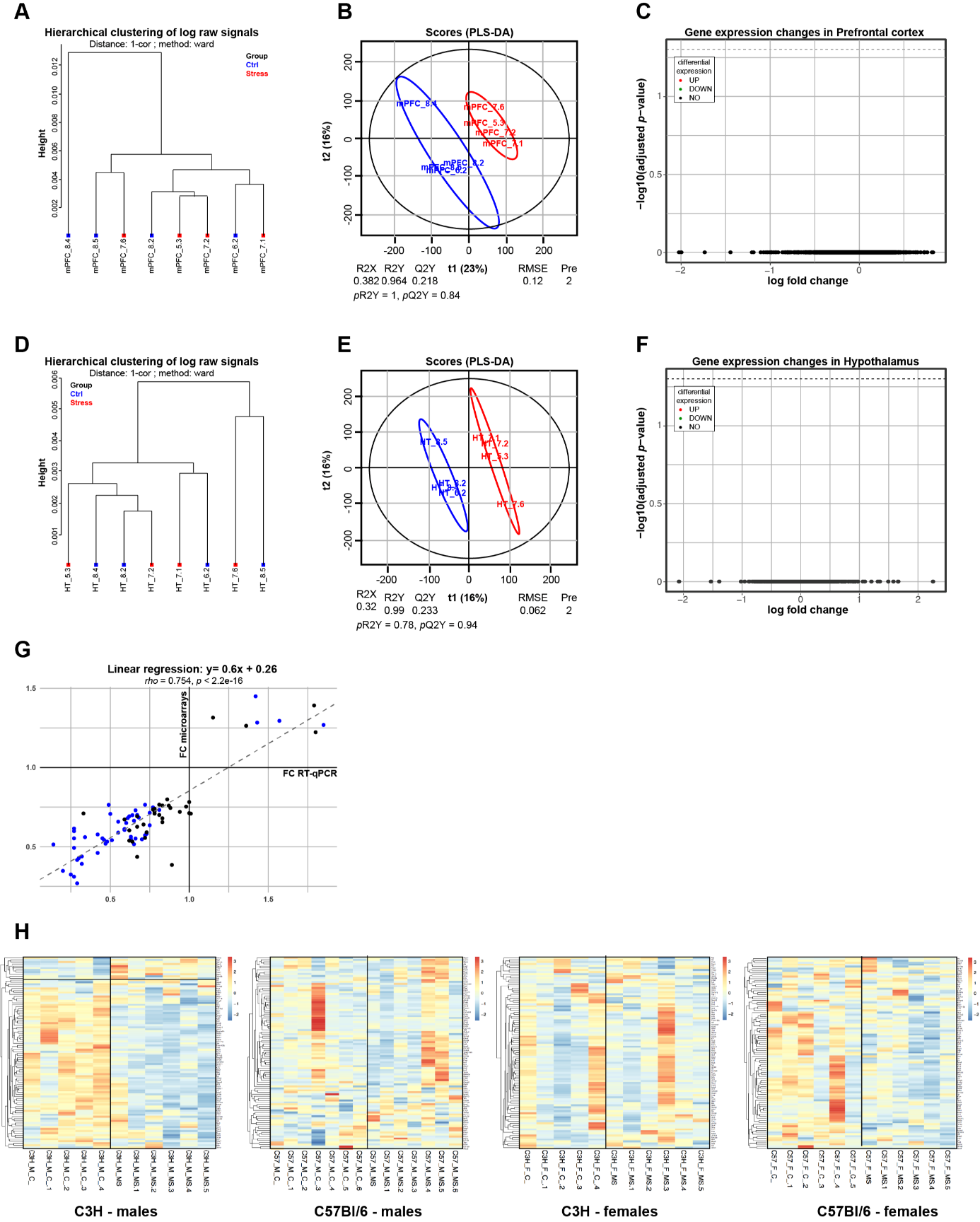
**

**Figure S2. Additional information concerning the transcriptomic analysis.** Clustering by Euclidean distance of the raw transcriptomic datasets from Control (blue) or MS (red) male adult offspring of mPFC **(A)** and HT **(D)**. Score plots from the PLS-DA classification into Control (blue) and MS (red) groups. A model is considered robust when the response variance explained (R2Y) is higher than the predictive performance of the model (Q2Y). A model with a Q2Y > 0.5 is considered to have a good predictive performance, which is not the case neither for mPFC **(B)** not HT **(E)**. Volcano plot depicting no significantly differentially expressed genes were found between the MS and Control conditions neither for mPFC **(C)** not HT **(F)**. n = 4 mice/group. A plot of the fold change (FC) calculated for RT-qPCR and microarray values between male MS and Control NAc for 84 genes. The RT-qPCR data correlated with the microarray data for most of the 84 FC, with a linear equation: y = 0,6x + 0.26 and a high correlation coefficient (rho = 0.754). Spearman’s test indicated that this correlation was highly significant (p < 2.2e10-16). More than 60% of the genes tested (51 out of the 84) were significantly differentially expressed (adj p < 0.05, blue dots) n = 4 mice *per* group **(G)**. Expression clustering for the 87 gene studied in the TLDA for C3H/HeN males (Control n = 5, MS n = 6), C57Bl/6J males (Control n = 7, MS n = 7), C3H/HeN females (Control n = 5, MS n = 6) and C57Bl/6J females (Control n = 6, MS n = 6).
