# Supplementary material for: Strain-specific changes in nucleus accumbens transcriptome and motivation for palatable food reward in mice exposed to maternal separation
Source: Front Nutr. 2023 Jul 26;10:1190392. doi: 10.3389/fnut.2023.1190392 (PMC10411197; doi:10.3389/fnut.2023.1190392)
Supplement: Supplementary file 1 [file Data_Sheet_1.zip › Supplementary Statistics.docx]

**FIGURE 4A: FIXED RATIO-1 (FR-1)**

**FOUR-WAY ANOVA WITH REPEATED MEASURES**

| **Effect** | **F** | **p-value** |
| --- | --- | --- |
| **Strain** | **F_(1,44)_ = 21.0610** | **p = 0.000037** |
| MS | F_(1,44)_ = 0.1421 | p = 0.707987 |
| Strain × MS | F_(1,44)_ = 0.3900 | p = 0.535541 |
| **Lever** | **F_(1,44)_ = 545.9653** | **p = 0.000000** |
| **Lever × Strain** | **F_(1,44)_ = 19.7954** | **p < 0.000058** |
| Lever × MS | F_(1,44)_ = 0.0016 | p = 0.967883 |
| Lever × Strain × MS | F_(1,44)_ = 0.4143 | p = 0.523148 |
| **Session** | **F_(4,176)_ = 64.1420** | **p < 0.000000** |
| **Session × Strain** | **F_(4,176)_ = 12.1069** | **p < 0.000000** |
| Session × MS | F_(4,176)_ = 0.6314 | p = 0.640763 |
| Session × Strain × MS | F_(4,176)_ = 0.5267 | p = 0.716276 |
| **Session × Lever** | **F_(4,176)_ = 78.6864** | **p < 0.000000** |
| **Session × Lever × Strain** | **F_(4,176)_ = 11.0936** | **p < 0.000000** |
| Session × Lever × MS | F_(4,176)_ = 0.6302 | p = 0.641564 |
| Session × Lever × Strain × MS | F_(4,176)_ = 1.1178 | p = 0.349697 |

**PLANNED COMPARISONS**

|  | **C57Bl/6J** | **C3H/HeN** |
| --- | --- | --- |
| **S1 – Active vs. Inactive lever** | **p = 0.000739** | **p = 0.008473** |
| **S2 – Active vs. Inactive lever** | **p = 0.000000** | **p = 0.000000** |
| **S3 – Active vs. Inactive lever** | **p = 0.000000** | **p = 0.000000** |
| **S4 – Active vs. Inactive lever** | **p = 0.000000** | **p = 0.000000** |
| **S5 – Active vs. Inactive lever** | **p = 0.000000** | **p = 0.000000** |
| **Active lever – S1 vs. S2** | **p = 0.000010** | **p = 0.000000** |
| **Active lever – S1 vs. S3** | **p = 0.000001** | **p = 0.000000** |
| **Active lever – S1 vs. S4** | **p = 0.000000** | **p = 0.000000** |
| **Active lever – S1 vs. S5** | **p = 0.000000** | **p = 0.000000** |
| **Active lever – S4 vs. S5** | **p = 0.000000** | **p = 0.000000** |
| **Inactive lever – S1 vs. S2** | **p = 0.023306** | **p = 0.007287** |
| **Inactive lever – S1 vs. S3** | **p = 0.012774** | **p = 0.000040** |
| **Inactive lever – S1 vs. S4** | p = 0.051948 | p = 0 085905 |
| **Inactive lever – S1 vs. S5** | **p = 0.007383** | p = 0.069618 |
| **Inactive lever – S4 vs. S5** | p = 0.470922 | p = 0.906805 |

|  | **C57Bl/6J vs. C3H/HeN** |
| --- | --- |
| **Active lever – S1** | p = 0.808423 |
| **Active lever – S2** | p = 0.516488 |
| **Active lever – S3** | **p = 0.000185** |
| **Active lever – S4** | **p = 0.000000** |
| **Active lever – S5** | **p = 0.000000** |

**FIGURE 4B: RANDOM RATIO (RR)**

**FOUR-WAY ANOVA WITH REPEATED MEASURES**

| **Effect** | **F** | **p-value** |
| --- | --- | --- |
| Strain | F_(1,44)_ = 0.0944 | p = 0.760139 |
| MS | F_(1,44)_ = 2.6207 | p = 0.112621 |
| Strain × MS | F_(1,44)_ = 2.3710 | p = 0.130772 |
| **Lever** | **F_(1,44)_ = 160.0448** | **p = 0.000000** |
| Lever × Strain | F_(1,44)_ = 0.0268 | p = 0.870700 |
| Lever × MS | F_(1,44)_ = 1.7048 | p = 0.198452 |
| Lever × Strain × MS | F_(1,44)_ = 2.2376 | p = 0.198452 |
| **RR** | **F_(2,88)_ = 28.8858** | **p = 0.000000** |
| RR × Strain | F_(2,88)_ = 0.8479 | p = 0.431774 |
| RR × MS | F_(2,88)_ = 0.3097 | p = 0.734487 |
| **RR × Strain × MS** | **F_(2,88)_ = 3.5840** | **p = 0.031890** |
| **RR × Lever** | **F_(2,88)_ = 28.7530** | **p = 0.000000** |
| RR × Lever × Strain | F_(2,88)_ = 0.4360 | p = 0.648029 |
| RR × Lever × MS | F_(2,88)_ = 0.2921 | p = 0.747444 |
| **RR × Lever × Strain × MS** | **F_(2,88)_ = 3.4639** | **p = 0.035640** |

**PLANNED COMPARISONS**

| **C57Bl/6J : Active vs. Inactive lever** | **p = 0.000000** |
| --- | --- |
| **C3H/HeN Control: Active vs. Inactive lever** | **p = 0.000009** |
| **C3H/HeN MS: Active vs. Inactive lever** | **p = 0.000000** |
| **RR5 Active lever: C57Bl/6J Control vs. C3H/HeN Controls** | p = 0.949948 |
| **RR10 Active lever: C57Bl/6J Control vs. C3H/HeN Controls** | p = 0.352800 |
| **RR20 Active lever: C57Bl/6J Control vs. C3H/HeN Controls** | p = 0.132125 |
| **RR5 Active lever: C57Bl/6J MS vs. C3H/HeN MS** | p = 0.225154 |
| **RR10 Active lever: C57Bl/6J MS vs. C3H/HeN MS** | p = 0.451807 |
| **RR20 Active lever: C57Bl/6J MS vs. C3H/HeN MS** | p = 0.309889 |

| **C57Bl/6J : RR5 vs. RR10** | **p = 0.000003** |
| --- | --- |
| **C57Bl/6J : RR5 vs. RR20** | **p = 0.000020** |
| **C3H/HeN Control: RR5 vs. RR10** | p = 0.070181 |
| **C3H/HeN Control: RR5 vs. RR20** | p = 0.236785 |
| **C3H/HeN MS: RR5 vs. RR10** | **p = 0.000509** |
| **C3H/HeN MS: RR5 vs. RR20** | **p = 0.000419** |

**FIGURE 4C: PROGRESSIVE RATIO (PR)**

**ANOVA**

| **Effect** | **F** | **p-value** |
| --- | --- | --- |
| **Strain** | **F_(1,42)_ = 9.7686** | **p = 0.0032** |
| MS | F_(1,42)_ = 0.8161 | p = 0.3715 |
| Strain × MS | F_(1,42)_ = 2.6177 | p = 0.1132 |
| **Time** | **F_(59,2478)_ = 141.0819** | **p < 0.001** |
| **Time × Strain** | **F_(59,2478)_ = 17.4244** | **p < 0.001** |
| Time × MS | F_(59,2478)_ = 0.4517 | p = 0.9999 |
| **Time × Strain × MS** | **F_(59,2478)_ = 2.6462** | **p < 0.001** |

**PLANNED COMPARISONS**

| **C57Bl/6J Control vs. C3H/HeN Controls** | **p = 0.001696** |
| --- | --- |
| **C57Bl/6J MS vs. C3H/HeN MS** | p = 0.292513 |
| **C57Bl/6J MS vs. C3H/HeN Controls** | **p = 0.292513** |
| **C57Bl/6J: Controls vs. MS** | p = 0.608146 |
| **C3H/HeN: Controls vs. MS** | p = 0.088244 |
